# Body Weight Estimation for Dose-Finding and Health Monitoring of Lying, Standing and Walking Patients Based on RGB-D Data

**DOI:** 10.3390/s18051311

**Published:** 2018-04-24

**Authors:** Christian Pfitzner, Stefan May, Andreas Nüchter

**Affiliations:** 1 Department of Electrical Engineering, Precision Engineering, Information Technology at the Techniche Hochschule Nürnberg Georg Simon Ohm; Keßlerplatz 12, 90489 Nuremberg, Germany; stefan.may@th-nuernberg.de; 2 Department of Informatics VII: Robotics and Telematics at the Julius-Maximilians University Würzburg, Am Hubland, 97074 Wuerzburg, Germany

**Keywords:** image processing, machine learning, perception, sensor fusion, segmentation, RGB-D, thermal camera, kinect, human body weight, stroke

## Abstract

This paper describes the estimation of the body weight of a person in front of an RGB-D camera. A survey of different methods for body weight estimation based on depth sensors is given. First, an estimation of people standing in front of a camera is presented. Second, an approach based on a stream of depth images is used to obtain the body weight of a person walking towards a sensor. The algorithm first extracts features from a point cloud and forwards them to an artificial neural network (ANN) to obtain an estimation of body weight. Besides the algorithm for the estimation, this paper further presents an open-access dataset based on measurements from a trauma room in a hospital as well as data from visitors of a public event. In total, the dataset contains 439 measurements. The article illustrates the efficiency of the approach with experiments with persons lying down in a hospital, standing persons, and walking persons. Applicable scenarios for the presented algorithm are body weight-related dosing of emergency patients.

## 1. Introduction

When it comes to the treatment of ischemic stroke patients, it is crucial to solve the blood clot in the brain vessel as fast as possible. For the treatment of ischemic strokes, the medicament rtPA was approved in 1996 by the U.S. Food and Drug Administration [1]. The medicine has to be given with a dosage of 0.9 mg per kilogram of the patient’s body weight. Furthermore, a maximum dose of 90 mg is specified for patients weighing more than 100 kilograms. It is best used within the first hour after the appearance of stroke symptoms. After three hours, side effects can prevail over the solving of the blood clot. Because of this narrow time window, physicians are in a hurry for treatment. Weighing a patient on a common standing scale is often not possible because the patient is in pain or is not able to stand due to other symptoms of stroke, e.g., paralysis. The obvious way to determine the body weight of someone quickly is to ask the person. However, if it comes to stroke, only half of the patients are knowledgeable and are not handicapped by stroke symptoms [2]. Additionally, elderly patients might suffer from dementia and cannot provide a reliable value for their body weight. Furthermore, relatives who could be asked might not be available in the trauma room or do not know the body weight of the patient. In addition, anthropometric methods exist, where lengths and circumferences of body parts are measured with a measuring tape. Based on an empirical equation, e.g., the equation for stroke patients presented by Lorenz et al. [3], gives an estimation of body weight. The disadvantage of those anthropometric methods is that the patient has to be moved and the measuring is time-consuming.

Therefore, visual estimation of the patient’s body weight by the attending physician in the emergency room has become routine worldwide. In a registry with 27,910 stroke patients, only 14.6 percent were weighed [4]. However, several studies [5,6,7] illustrate that such a weight estimation by a visual guess from a physician is often not sufficient for dosing: Every third patient receives a dosage out of the ±10 percent bound. This result can be improved if the average estimation of several persons from medical staff is taken. Furthermore, the estimations from nursing staff are more reliable than the visual guesses by physicians [2].

The observation of body weight is also essential in elder care: People with a healthy weight can recover better from sickness than people who are underweight or obese. However, older people often have a reduced appetite, coupled with a decline in biological and physiological functions [8]. In elder care, people are weighed on common standing scales to observe changes in body weight. Multiple approaches with 3D sensors are being tested in the field of elder care, especially since the release of the low-cost Microsoft Kinect camera [9]. Some applications of these approaches are fall detection or the monitoring of breathing [10,11]. The contact-less body weight approach can be combined in context with these other approaches to monitor changes in body weight to improve elder care.

In contrast to the scenario of patients being measured on a stretcher, the weighing of standing people can be easily done on a spring scale. However, the automatic weighing of several people in a short time can bring a benefit in some applications: Since 2017, the Finnish airline Finnair weighs passengers to determine the total weight of an airplane for take-off. While the weight of baggage is measured with a scale, the weight of the passengers is only roughly rated with standardized weights [12]. The precise knowledge about the weight gives possibilities to optimize fuel requirements and therefore operating costs [13]. In 1985, a McDonnell Douglas DC-8 jetliner crashed with 256 people on board. One reason for the crash might have been the underestimated onboard weight, which was mentioned in the occurrence report [14]. Furthermore, the motivation for a visual weight system is gained as objects that the subject is wearing or carrying, e.g., a backpack, can be filtered out for weight estimation.

The presented approach is an extension of Pfitzner et al. [15]: While this previous work had the clear focus on clinical use, the work presented here extends the approach towards standing and walking people. First, this article contributes a summary about the visual body weight estimation for various situations. The settings for the different approaches are compared, as well as the results of the experiments. Second, the article shows that the feature set from previous work is also suitable for body weight estimation of standing or walking subjects. To obtain the body weight of walking subjects, a clustering method is presented, combining the estimations from each frame, to a single and also a more robust estimation. Finally, the article provides the 3D data used for experiments so that other research groups can contribute to this topic.

The paper is structured as follows: First, the related work concerning the body weight estimation based on a camera system is presented, focusing on lying, standing, and walking people. Second, the here applied and published dataset for body weight estimation based on RGB-D-T (color, depth and thermal) data is explained. In the following section, the approach for body weight estimation is presented and separated for standing and walking persons. Experiments with the here applied dataset and a dataset from related work demonstrate the efficiency of the developed algorithm. The results are examined in comparison to other approaches for visual weight estimation from related work. Finally, the paper concludes with a discussion and plans for future work.

## 2. Related Work

The related work is subdivided for lying, standing and walking people and further provides a summary of weighing and estimation devices for clinical usage.

### 2.1. Common Weighing and Medical Estimation Devices

Scales come in a wide range. The most common type is standing scales. Analog scales use a reference weight or a spring to obtain the body weight, while modern digital scales use strain gauges and a change in resistance to get a value for body weight. In the clinical scenario, chair scales exist, so a patient does not need to stand for the process of weighing. Different types of bed scales are available to weigh patients who are lying down. First, scales can be designed as a single plate integrated into the floor where the bed is placed. Second, bed scales are available with multiple weighing devices, which are attached to each wheel. The sum of all weight is the total weight of the bed, including the patient. In both scenarios, the tare weight of the empty bed has to be known. Consequently, either the bed is weighed in advance without the patient or the tare weight of the bed has to be identified. Choosing the wrong type of bed can result in a high degree of error concerning the patient’s weight. Furthermore, different attachments, such as medical devices or handrails, can cause a change in tare weight. In addition, it is possible to integrate multiple strain gauges directly into the mattress. This solves the issue of determining the tare weight of the bed. It is also possible to integrate weighing directly into the computer tomography to speed up the process of weight acquisition [16].

Furthermore, rulers exist to approximate body weight for medical usage: Approximation rulers are common to estimate the body weight of young children; the Broselow tape was developed in 1985 by James Broselow and Robert Luten. It provides nine different weight groups for children younger than 12. A colored scale on the measuring tape relegates to different medical sets, prepared for emergency treatment of the different weight groups in case of an emergency. Several studies illustrate that the Broselow tape is reliable for first aid personnel [17]. However, for children, the estimation of the parents can be even more reliable, if available [18].

### 2.2. Estimation from Lying People

The body weight estimation of lying people is important mainly in the scenario of clinical usage. Most patients are already lying on a stretcher or a bed. Furthermore, the here presented approaches are suitable for bedridden patients.

Pirker et al. [19] employed 16 stereo cameras around a stretcher. Additional projectors are needed for complete illumination. A parametric human model complements the back side of the body. Composed images are filtered for noise reduction and, finally, the volume is calculated with the help of cross-sections along the body. Because of the high amount of cameras around the patient’s bed, physicians would be constricted during treatment.

The here presented algorithm for the estimation of lying, walking and standing people is the continuation of preceding work: In 2015, Pfitzner et al. [20] showed an approach for body weight estimation with a depth camera. The algorithm extracts only the volume of a subject lying on a medical stretcher, multiplying it with a fixed value for the density. Color and depth gradient achieve the segmentation. The focus of this application was set on the body weight estimation of stroke patients within the treatment process in the trauma room. Although the approach is straightforward, the system was more reliable than the visual guess performed by the medical staff: 79 percent of all patients received a sufficient body weight estimation, while the visual guess from a physician could only provide a sufficient estimation in 68 percent of patients.

**Figure 1:**
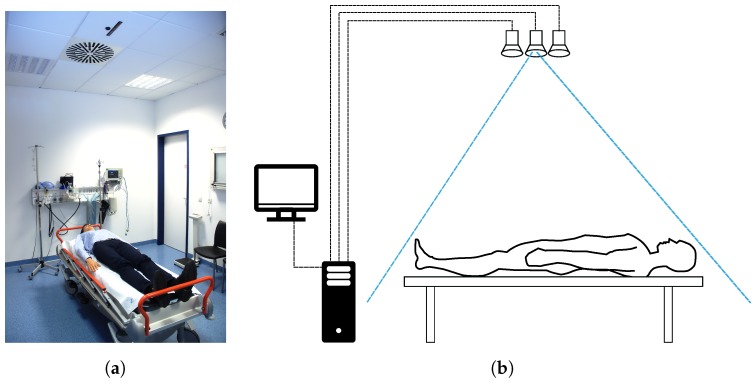
Clinical integration of sensors into the trauma room, as shown in [15]. Within the scenario of trauma room in which physicians mostly treat emergency patients, the sensor system is integrated into the ceiling (**a**); The system does not hinder the physician while treating the patient, who is often lying on a medical stretcher. Besides the sensors in the ceiling, the system consists of a computer system—including a keyboard and a mouse for interaction, a monitor for visualization and a barcode scanner to identify patients with their ID (**b**). The connection between the sensors and the computer is achieved by USB cables. (**a**) Trauma room with sensors integrated into the ceiling; (**b**) Schematic of the sensor system and its connections to a computer.

Figure 1 shows the scene in the trauma room with a patient on the stretcher and the complete system, as presented in Pfitzner et al. [20]. The setup with the patient lying on a medical stretcher and the sensors integrated into the ceiling is the same as in the following previous work.

The approach was extended in 2016 by the work of Pfitzner et al. [15]. An additional thermal camera improves the segmentation, and the patient in the fused field of view can be clearly segmented from the mattress that the subject is lying on. Furthermore, this paper introduced an extended feature extraction, as well as a machine learning approach—here an ANN—to improve the outcome in body weight estimation, by minimizing outliers and improving the standard deviation for the relative error. In total, 89.9 percent of all subjects received an estimate of ±10 percent. For this approach, a patent exists [21].

Pfitzner et al. [22] presented a comparison of different depth sensors for the scenario of body weight estimation. In conclusion, the Kinect One can provide better results in body weight estimation—95.3 percent for the ±10 percent range—compared to the estimation based on the data of the Kinect with 94.8 percent. Additionally, this work also presents a correlation analysis of the extracted features, and how a different configuration of the available features can provide a reliable result.

### 2.3. Estimation from Standing People

In contrast to the estimation of lying people, the scenario for standing or walking people is more complex: The person is not aligned to a fixed surface on the back. Furthermore, the posture and the position of the subject changes in a sequence of frames.

Robinson and Parkinson [23] developed an approach for the body weight estimation of standing people. Here, anthropometric features are extracted from a scene’s point cloud and the raw sensor data from an RGB-D sensor with a person standing in front of it can be seen. This approach also demonstrated that these raw features from the point cloud could lead to a bias because of un-calibrated sensor or noise. Furthermore, even thin clothes can confuse the extraction of the features, like the circumference of a body part, e.g., waist or hip circumference.

Cook et al. [24] presented a framework based on a structured light sensor for radiation dose estimation in CT examinations. In preliminary experiments, they showed results for five persons standing in front of a structured light sensor. The measured volume of the patient differs due to different positions of their arms.

With the help of skeleton tracking, Velardo and Dugelay showed a computer vision system to prove the health of a person with the help of a structured light sensor [25]. Apart from sensing the age of the subject, the sensor measures anthropometric features from arms, legs, and the body. The authors provide a trained statistical model from a medical database, containing anthropometric measurements from more than 28,000 subjects, as well as the ground truth body weight. This approach has the benefit of a large sample size for training. However, the estimation of the anthropometric features based on the RGB-D data is hard due to the sensor’s noise. Additionally, the system provides information about obesity and nutrition to the user.

Based on a side-view feature, Nguyen et al. [26] developed a method to estimate the human body weight of standing people captured with an RGB-D camera. A model is trained based on regression. Together with handling the gender data, the algorithm can reach the mean average error of 5.20 kg over 300 subjects. In an additional experiment, the authors proved that the body weight estimation based on RGB-D data is more reliable compared with the human estimation. Furthermore, the utilized dataset with 300 subjects is published as an open-access source, containing the RGB-D data, the ground truth gender, and the ground truth body weight. This dataset is also applied to the following experiments.

### 2.4. Estimation from Walking People

Beside the body weight estimation from a single frame, it is also possible to estimate it by a sequence of sensor data. Labati et al. [27] developed a body weight estimation suitable for walking persons. The focus was set on a contact-less and low-cost method. The method is based on frame sequences from two cameras, which are placed to get a frontal and a side-view of the walking person. The feature vector consists of the height of a person, an approximation of the body volume, an approximation for the body shape and the walking direction. The extracted features are forwarded to an ANN to obtain body weight. Experiments are performed with 20 subjects, walking in eight different directions. A maximum absolute mean error was recorded with less than 2.4 kg.

Arigbabu et al. [28] demonstrated the extraction of soft biometrics, e.g., body height and weight, based on video frames from a single monocular camera. Due to a homogeneous background, the people’s silhouette can be extracted easily with state of the art image processing techniques like background subtraction. The silhouette is converted into a binary mask, where 13 features are extracted depending on the pixel density in segmented regions. The feature vector is finally forwarded to an ANN to estimate the body weight. In experiments with 80 subjects, they reached a mean average error of 4.66 kg the estimation of body weight. The update rate of the extraction of all described soft biometrics was about 1 Hz. The approach was compared with the previously presented approach by Labati et al. [27] and Velardo and Dugelay [25].

Most of the approaches presented here use neural networks as a machine learning approach to generate a model for body weight estimation. The difference in the approaches can be found in the types of features forwarded to a neural network. In contrast to related work, the approach in this paper is not limited to a particular application. The selected features for machine learning are suitable for the scenarios of subjects who are lying, standing or walking. They can be used in general for the estimation of body weight. Furthermore, Table 1 compares the results of related work as a summary. The approaches presented by Nguyen et al. [26], Velardo and Dugelay [25], Labati et al. [27] and Arigbabu et al. [28] are compared in the experiment section.

## 3. Approach for Visual Body Weight Estimation

**Figure 2:**
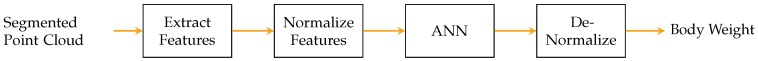
Process of body weight estimation.

The algorithm is subdivided into sections for sensor fusion, segmentation, and feature extraction. It leads to a learning approach based on an ANN to obtain the body weight of a single person. Figure 2 illustrates the procedure in body weight estimation based on the previously segmented point cloud.

### 3.1. System Description

The system uses different sensors, depending on the recorded dataset. It was developed for previous work [15,20] to be integrated into the clinical environment. There the system includes a Microsoft Kinect, a Microsoft Kinect One and an Optris PI400 thermal camera. A single depth sensor is sufficient for body weight extraction. However, the developed algorithm should not depend on the applied sensor. Therefore, experiments are performed with different sensors. Table 2 compares the sensors to each other.

Both the Kinect and the Kinect One are RGB-D cameras providing a color stream RGB, and a depth per pixel D. The first Kinect camera was released in 2011 bringing a low-cost consumer product into robotics. The sensor brought multiple applications and made an impact well beyond the gaming industry [29]. The Kinect holds a sensor for infrared (IR) and a sensor for color. Both sensors are calibrated to each other. The structured light principle obtains depth: A projector emits a known pattern in the environment. This pattern is seen by the IR sensor from a different pose to calculate the depth for an arbitrary pixel. Khoshelham and Elberink [30] illustrate the sensor’s characteristics in image quality and noise.

In contrast to that, the Kinect One works by the Time-of-Flight (ToF) principle [31]: Having a highly precise measurement device for the time, it would be possible to calculate the distance between a light source and an object by measuring the time. The range of a given point can be calculated by the time *t* the light travels with the help of the speed of light *c* with d=0.5·t·c. Due to the fast traveling light, the distance measurement is obtained by modulated light: A source emits a light pulse towards an object. The frequency for modulation is known, and a phase shift can be measured from the reflected signal.

The here applied depth sensors differ not only in their resolution, but also the different principle provides a diverse characteristic of depth. Both sensors are compared to each other by Sarbolandi et al. [32]. Today, there exist various types of RGB-D sensors, which are suitable for the body weight estimation approach, e.g., Asus Xtion cameras from the Intel RealSense series [33]. The thermal camera is state of the art and is added to the sensor set to ease segmentation based on a simple thermal threshold. In this presented sensor configuration, the thermal camera is the most expensive part. It was used because it was already available from an earlier project. However, a cheaper thermal camera with a lower resolution and frame rate can be used for the segmentation. Pfitzner et al. [20] illustrated that the visual body weight estimation is possible without a thermal camera, but outliers due to insufficient segmentation can occur.

The algorithm—including the sensor fusion, the feature extraction and the forwarding to an artificial neural network—is implemented on a conventional desktop computer, which is installed in the trauma room. The computer in the trauma room, which is equipped with an Intel i7 of the 4th generation, can provide the result in body weight estimation within 300 ms, including the saving of the sensor data. The software does not rely on specialized hardware, like a high-end graphics card, although the processing speed could benefit from parallelization. For offline processing, a mobile computer (Dell M4800) is used, having a maximum power consumption of less than 80 Watt  [34]. Therefore, the complete hardware could be designed with less than 100 W, including the mobile computer, the thermal camera (<2.5 Watt) and the Microsoft Kinect (12 Watt). Table 3 illustrates that the processing time for the desktop computer and the mobile computer is similar. A small experiment in our laboratory showed that the approach is also suitable for small size embedded computers, e.g., a Raspberry PI. With a reduced visualization, and without the saving of the sensor’s data to the database, this configuration provided the estimation of body weight in around 5 s, see Table 3. The system is then limited in real-time visualization, as well as process time, and the estimation of the body weight is available with a higher delay. However, the embedded computer can have the benefit of lower power consumption and a smaller footprint, which provides easier integration in the clinical environment.

### 3.2. Sensor Fusion

All applied projective depth, color, and thermal sensors are calibrated intrinsically based on the method presented by Zhang [35]. Therefore, a single calibration pattern is used, which is visible in depth, color, and thermal frame. Gonzalez-Jorge et al. [36] present different types of suitable calibration targets. The here applied calibration target consists of a metal plate on the back which is colored white and a black wooden plate on top. The wooden plate has holes in a circular pattern. The metal plate can be heated. Because of a space between the metal and the wood plate, a thermal gradient is visible, and the wholes appear to be warmer than the top surface. The circle pattern is therefore visible in the spectrum of the thermal camera [15].

**Figure 3:**
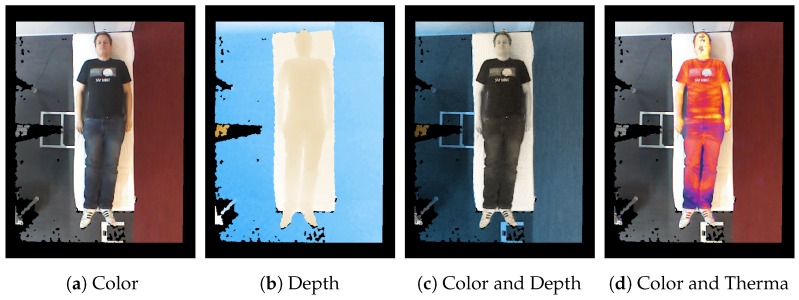
Visualization of sensor fusion: Figure (**a**) shows the raw color stream from an RGB-D camera. The depth stream can be visualized using a colormap, here drawing blue values for far objects and drawing white and orange for nearer objects (**b**). The stream from the thermal camera can be visualized in several ways: either it is drawn with false-color representation (**c**) or can be combined with other streams—here a combination of the color stream, highlighted with temperature (**d**). The here presented image-based sensor fusion is achieved by intrinsic and extrinsic camera calibration, which is presented in Figure 4.

The results of sensor fusion can be displayed in different settings. Besides the typical representation on the screen as a color image of the scene, the depth can be visualized by a color mapping. Furthermore, it is also possible to illustrate the scene as a false-color representation for temperature or fused with the color stream, similar to that presented by Vidas et al. [37]. This is achieved by comparing the color channel of every point in the cloud. Figure 3 illustrates the sensor fusion and its visualizations: In Figure 3c, the data from the color sensor of the Kinect camera is fused with its depth stream. In the fused image, the color stream provides the intensity of each pixel as a grayscale, while the color of a pixel arranges the depth in the scene, as shown in Figure 3b. In addition, the color data and the thermal data are aligned to be visible at the same time, see Figure 3d. From the given data, further data can be calculated to enhance the point cloud or the depth image, e.g., with normals.

**Figure 4:**
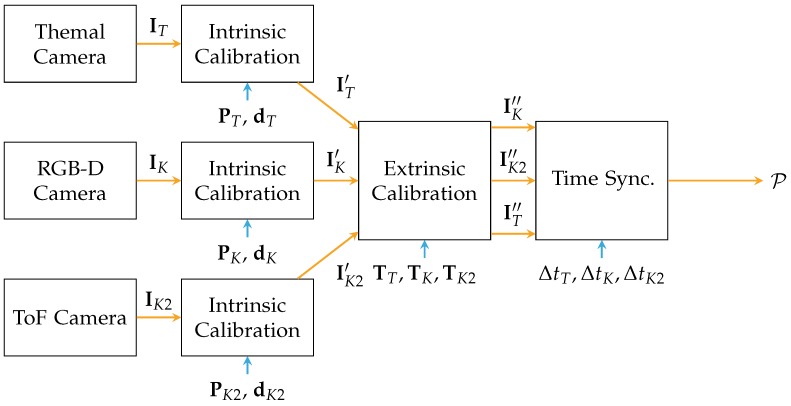
Process of sensor calibration: First, all projective sensors are calibrated intrinsically to remove distortions from the image and to obtain the projection matrix for each sensor $P_{T}$, $P_{K}$ and $P_{K2}$. Furthermore, the coefficients for distortions $d=(k_{1}\;k_{2}\;k_{3}\;p_{1}\;p_{2})$ are necessary for rectification. The vector contains the parameters for radial distortion ($k_{1},k_{2},k_{3}$), as well as the parameters for tangential distortion ($p_{1}$,$p_{2}$) [35]. Second, the sensors are calibrated extrinsically, estimating the transformations between the sensors T. The calibrated images are noted by $I_{T}^{\prime }$, $I_{K}^{\prime }$, and $I_{K2}^{\prime }$. Finally, the data from the sensors are synchronized in time based on $\Delta t_{T},\Delta t_{K},\Delta t_{K2}$. The synchronized images are noted by $I_{K}^{\prime \prime }$, $I_{K2}^{\prime \prime }$, and $I_{T}^{\prime \prime }$. After this process of calibration, sensor fusion can be applied and data is converted towards a Cartesian point cloud $P\in R^{3}$ .

Figure 4 presents the process of calibration for sensor fusion: The frames from the sensor are differentiated by indices, *K* for the Kinect, K2 for the Kinect One and *T* for the thermal camera. All three sensors are calibrated intrinsically. First, the raw streams from the sensors I are forwarded to rectification based on the determined intrinsic parameters P and d [35]. Second, the rectified images I are then calibrated extrinsically based on the previously estimated transformations T. Third, the aligned data I is synchronized in time by the method presented by Lussier and Thrun [38] with $\Delta t_{T},\Delta t_{K},\Delta t_{K2}$. Finally, a point cloud $P=(p_{1}\;p_{2}\;\ldots \;p_{n})$ containing *n* points, can be generated with the help of the pinhole camera model.

The intrinsic calibration aims to remove the aberrations from the lens, bringing the image in the form of the pinhole camera model. For the intrinsic calibration, the projection matrix P has to be determined. The matrix contains the focal length , as well as the offset to the sensor’s center. Therefore, based on the pinhole camera model, a point $p={\left(x\;y\;z\right)}^{T}\in R^{3}$ can be projected onto the sensor as a pixel $q={\left(u\;v\right)}^{T}$. For the extrinsic calibration, the world frame’s origin is set the same as the origin of the infrared sensor of the Kinect. The extrinsic factory calibration of both Kinect cameras is left as it is. The transformations between the two Kinect cameras and the thermal camera is estimated with the help of the same calibration pattern.

**Figure 5:**
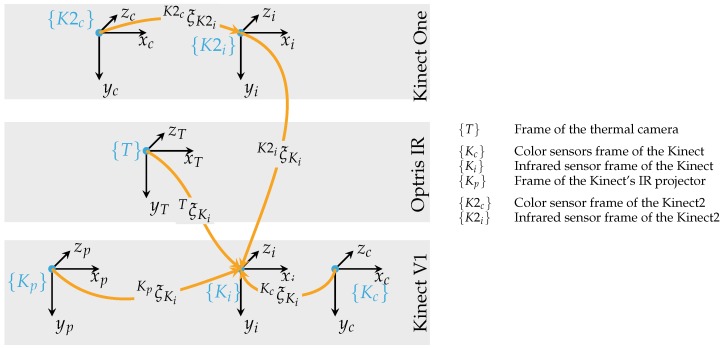
Transformation tree for the system’s sensors: The infrared frame of the Kinect is used as reference for the world coordinate origin {0}. The manufacturer already calibrates the 3D sensor’s own sensor frames. To obtain the transformation between the Kinect V1 and Kinect One, the IR sensors from both cameras are taken as a reference.

Figure 5 illustrates the transformation between the sensors. The extrinsic parameters—the rotation R and the translation t —are combined to a pose ${}^{A}\xi_{B}$ describing the relative pose of {B} with respect to {A}. After sensor fusion, every point can contain the Cartesian coordinates (x,y,z), color (rgb) and thermal data (*t*) with $p={\left(x\;y\;z\;rgb\;t\right)}^{T}$. For calibration and sensor fusion, OpenCV was applied [39].

### 3.3. Segmentation

The process of segmentation differs with the scene: A patient lying on a medical stretcher with physicians on his side is harder to segment than someone standing in an empty room with a clear distance to the wall behind him. The point cloud P is segmented in a set belonging to the person $P^{p}$ and a set for the environment $P^{E}$ with $P=P^{E}+P^{p}$. Therefore, a point can only belong to the person’s point cloud, or the environment. The segmentation for clinical applications is described by Pfitzner et al. [15]. For the reader’s convenience, it is also presented as follows: The patient is placed in a set range within the field of view (FOV) of the sensors mounted on the ceiling. This range is visible with markers on the floor. In an initial step, the amount of data in the point cloud is reduced. Therefore, the floor and all data outside the range of the markers on the floor is removed from the point cloud. After this step, the point cloud should contain mostly the patient and the stretcher he or she is lying on. Based on the available thermal data from the thermal camera, the segmentation can be done with a threshold in temperature. Points having a higher temperature than a fixed limit are included in the patient’s point set $P^{p}$. Physicians or family members close to the patient can be removed by finding the most significant contour easily under the assumption that the most significant part of the remaining scene is the patient and the stretcher. Based on the Random Sample Consensus (RANSAC) algorithm [40], the surface of the stretcher is obtained with a model for a plane. On one side, this is necessary to improve the outcome of segmentation. On the other side, the surface of the stretcher is necessary for the upcoming feature extraction. Morphological operations like erosion and dilation improve the outcome of segmentation [41]. Finally, the scene’s point cloud P is segmented, and the patient’s point cloud $P^{p}$ is available. To check if a patient is within the FOV of the camera, state of the art algorithms like the histogram of oriented gradients can be used [42]. Further, the measurement can be started by the medical staff by pressing a button attached to the wall in the trauma room. The segmentation in this medical scenario is reliable and robust. The data from the thermal camera provides good results in segmentation, sufficient for feature extraction. However, also without a thermal camera, the segmentation can be achieved, but outliers can occur, as illustrated in previous work [20].

**Figure 6:**
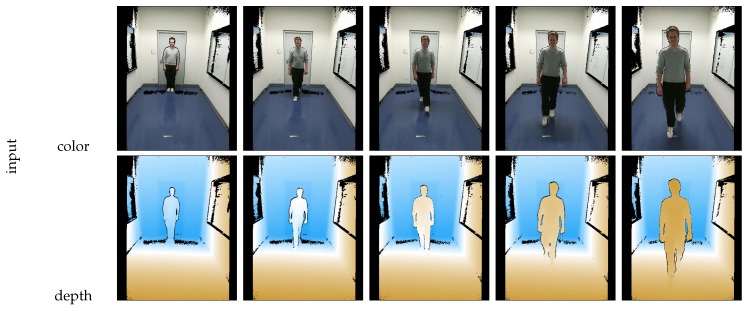

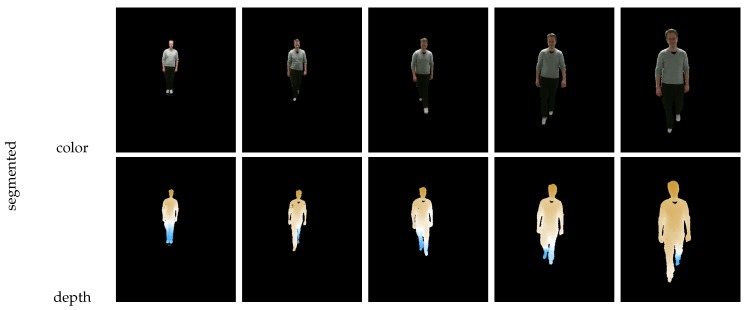
Sequence of someone walking towards the camera: The first two rows in the table illustrate the raw scene in color and depth representation, while the lower part of the table shows the segmented person. The sequence was recorded over four to five seconds. The scene is recorded with the Kinect One.

The segmentation of a standing or walking person is less complex: To segment the person from the background, a reference frame $P_{\mathrm{ref}}$ without the person is recorded in advance. The current frame containing the person is subtracted from the reference frame $P^{P}=P-P_{\mathrm{ref}}$. Due to the sensor’s noise, a threshold in distance should be applied to get a good outcome of background subtraction. Furthermore, to improve the outcome of the segmentation on the floor, the RANSAC algorithm can be applied to detect points on the floor and remove them from the scene’s point cloud. Therefore, the segmentation of the feet gets more accurate and robust. Outliers and jumping edge errors can be removed by morphological filters or statistical outlier filters. Figure 6 illustrates the segmentation based on background subtraction with a person walking towards the camera. This procedure is similar as presented in related work by Labati et al. [27] and Nguyen et al. [26].

### 3.4. Feature Extraction

Based on the segmentation, features are obtained from the person’s point cloud $P^{P}$. The position of a patient does not vary much in the clinical scenario with the patient on a medical stretcher in a previously defined position of the bed and in a fixed distance from the sensors. In contrast to that, the pose of multiple persons standing in front of a camera can vary more; while walking the pose of the person changes from frame to frame. Therefore, it is necessary that the extracted features are robust against changes in scale, translation, and perspective. The difference in posture is small for most people standing in front of the camera or lying on a stretcher: most of them have their arms aside their body and a few have their arms crossed over their stomach.

The extracted features are presented in Table 4. The correlation of those features to the ground truth body weight is shown in Pfitzner et al. [22]. A good feature is invariant against scale (s), rotation (r), translation (t), perspective (pe) and posture of the person (po) in front of the camera. However, while most of the here presented features are invariant for scale, due to the applied 3D data, no feature is invariant against changes in posture. Therefore, the data applied for training the model should cover many different common postures for standing and walking people.

The features can be grouped: The features $f_{1}$ to $f_{4}$ are simple geometric features. The estimation of the volume is only possible due to the stretcher the patient is lying on. The volume is calculated based on a triangle mesh of the person’s frontal surface *s*. The not visible surface on the back of the person is modeled by a single plane. The calculation of the volume is presented in detail in Pfitzner et al. [20]. Further, the triangle mesh is taken to calculate the frontal surface of a person. Although both features, the volume, and the surface, are only estimations and can be far from ground truth values, they can be a hint for an estimator: A person having a higher value for volume tends to be heavier compared to someone having a lower volume value. In addition, this first feature group contains the number of points belonging to the person’s point cloud $|P^{P}|$ and the calculated density of the scene, setting the number of points from the person in relation to the number of points of the whole scene |P|.

The second group of features ($f_{5}$ to $f_{10}$) is based on eigenvalues and the eigenvalues itself: The normalized eigenvalues have the benefit that they are invariant against coordinate transformations like scale, rotation, and translation. Therefore, the features based on these eigenvalues—sphericity, flatness, and linearity—are also invariant against transformations.

The third group consists of features from statistics: Compactness and kurtosis are normalized and therefore invariant against scale, rotation, and translation.

Features from the silhouette of a person are grouped in the fourth section: The area and length of the contour and the convex hull are invariant against rotation, and translation, but not against scale. However, a small change in posture can change the outcome from the calculation of contour and convex hull.

Related work showed that the body weight estimation could be improved if the gender is known [26]. The gender was here taken from ground truth, but could also be estimated by algorithm [26,43]. Apparently, the gender does not change in any way with applied transformation. Further, the cited algorithms are robust in detecting gender [43].

Table 5 demonstrates the changes in the feature values with different postures: The first scene shows a subject standing straight with the arms aside. The features are listed and calculated by the previously presented equations. In the second scene, the subject raises both hands a bit. The values for surface and density do not change much. In addition, the first eigenvalue nearly stays the same. However, the value for the third eigenvalue changes, due to the arms raised in front of the person. Flatness and sphericity—which correlate with the third eigenvalue—also change significantly. Compared to the third scene, where the subject stands with legs apart, the second eigenvalue changes most. Therefore, flatness and also linearity change. Comparing the first and the fourth scene, the subject crosses the arms: The surface lowers, as well as all features from contour and convex hull. The second eigenvalue lowers while the third eigenvalue increases. In the last scene, the subject is wearing a backpack. Comparing the features from this scene with the first scene, most of the features are within the same range. However, there are differences due to slight differences in posture. Apparently, the body weight estimation can ignore such objects as backpacks, if not visible to the sensor.

Concerning all the poses presented here, the features from contour and convex hull are able to vary the most: A subject having the arms aside can cause a much higher length in contour when there is a small gap between the body and the arm. However, as shown in [22], the length and area of the contour correlate with the body weight and therefore it can be useful to enclose such features for body weight estimation.

Machine learning minimizes the invariances in selected features. However, a suitable set for training and testing should cover most of the various poses, especially when the subject is moving during body weight estimation.

### 3.5. Weight Estimation Based on a Single Frame

The previously extracted features are forwarded to an ANN. The network is designed as a three-layer feedforward network, having one layer as input, one hidden layer, and a single output layer. The output layer consists of a single neuron representing the body weight in kilograms. The number of input units is set by the number of features forwarded to the network. For every element of the feature vector, an input unit exists.

The network is trained with a subset of the available data. For the upcoming experiments, 70 percent of a dataset is forwarded to the neural network for training. The remaining 30 percent of each dataset is used to evaluate the network. Those data are never used for training so the network cannot overfit for the training data. Learning is achieved by resilient propagation [44]. Regularization is applied with weight decay to improve the outcome. First, the error of training and testing decreases. After a while the error in testing dataset increases while the training error is still decreasing. This is the moment to abort the training to prevent an over-fitting. Due to randomized starting points, the learning via the neural network approach can come to different solutions for every trial.

### 3.6. Estimation of a Sensor Stream

**Figure 7:**
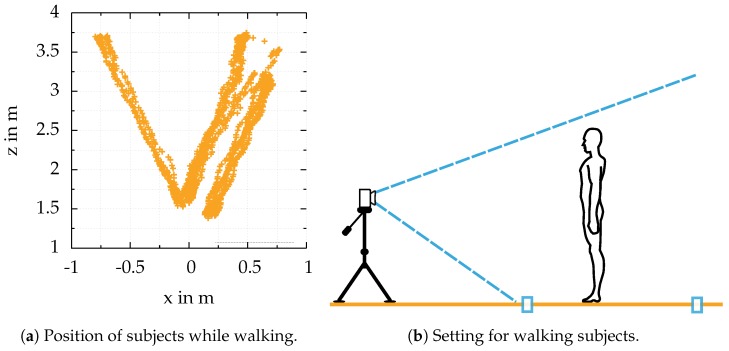
Poses of people walking towards the camera (**a**): The complete datasets consist of several independent experiments. Therefore, the poses of the people walking differ, depending on the orientation of the camera. The people stand at the first marker (**b**). While walking towards the second marker close to the camera, every frame from the sensor is saved for offline processing. The recording is stopped when the second marker is reached.

The FOV, the person’s height, and the maximum distance for 3D data acquisition mark the starting and end markers on the floor, see Figure 7b. Figure 7a illustrates the poses of all people walking towards the camera. Due to different settings for the experiments, the path people tend to walk differs. Further, the camera did not always have the same orientation towards the floor and was not always mounted at the same height.

First, the person is segmented from the background by the methods described in the previous section. Second, for every frame of the dataset, the body weight estimation is applied. In a scatter plot together with the ground truth body weight, a line becomes visible for every single person. Some of the estimations are close to the ground truth body weight. Even outliers of more than 30 percent occur. Therefore, taking an arbitrary frame from a person’s dataset will likely lead to a close to random result. Third, a clustering method is applied, so not only an arbitrary frame from a person’s dataset provides an estimation of the body weight. A Euclidean clustering method is applied to improve the outcome. The clustering is applied as follows: A dataset of a person D consists out of *N* frames from the sensor $D_{0},D_{1},\ldots ,D_{N}$. Every frame consists of a point cloud P.

1. For every frame in the dataset $D_{i}\in D$ estimate the body weight based on the calculated features $w_{i}\left(D_{i}\to f\right)$.
2. Calculate the mean distance $\bar{d}$ for every estimation of a dataset D to all other estimations by
  (1) $${\bar{d}}_{i}\left(D_{i}\right)=\frac{1}{N}\sum_{j=1}^{N}\left|w_{i}-w_{j}\right|\;\mathrm{where}\;i\neq j$$
  and store the calculated average distances in a vector $\bar{d}$.
3. Sort the calculated distances in an ascending order ${\bar{d}}_{0}\leq {\bar{d}}_{1}\leq \ldots \leq {\bar{d}}_{N}$.
4. Remove values with the highest distances. Keep a fixed amount of distances, e.g., 20 percent.
5. Calculate the centroid of the remaining estimations containing $n_{0.2}=0.2\cdot N$ estimations
  (2) $$\bar{w}=\frac{1}{n_{0.2}}\sum_{i=1}^{n_{0.2}}w_{i}$$
  which is the result of the body weight estimation based on a stream of data.

The principle in clustering is demonstrated in the upcoming section with experiments.

## 4. A Dataset for Body Weight Estimation

In addition to the here presented algorithm, a dataset is published to boost research in this field. Public datasets, as provided by Nguyen et al. [26] help to improve models for body weight estimation. Furthermore, developed algorithms and models can be applied to the dataset to generate comparable results. Depending on the recorded dataset, different sensors are used for recording. First, the Microsoft Kinect camera from the first generation of the XBox is used to obtain 3D data from the environment. Another sensor used for data acquisition is the second generation Kinect camera, the Kinect One. Additionally, a thermal camera is added and fused to the 3D data. This should ensure an easy segmentation approach based on a thermal threshold.

Table 6 illustrates the characteristics of the subjects in the dataset. The datasets are the following:

- **HospitalNoThermo**: From May 2014 to September 2014 a dataset was recorded from the Universitätsklinikum Erlangen, Germany, for preliminary testing. In this early dataset only RGB-D data is available without thermal data. The thermal camera was added after this experiment. The dataset contains 192 measurements.
- **Hospital**: This dataset includes feature values from trauma room patients from the Universitätsklinkum Erlangen, Germany. The dataset contains 127 measurements from people lying on a medical stretcher, recorded with a Microsoft Kinect. For this dataset a proper distribution is achieved consisting of people of different ages, body weights and shapes, see Table 4. Additionally, this dataset contains the patients’ self-estimation, age, sex, as well as anthropometric features like body height, abdominal girth, and waist circumference. The distance between the sensors and the subjects was around 2 m.
- **Event**: The features from this dataset were recorded at a public event, called Long Night of Science in 2015 in Nuremberg, Germany. People in this dataset were visitors of the public event. This dataset contains 106 people. For this public event, it was not convenient to take anthropometric measurements. Ground truth was validated with a standard digital scale. The dataset consists of sensor values from Kinect and thermal camera. Additionally, this dataset includes point clouds from Microsoft Kinect One.
- **Walking**: Based on the results of the previous three datasets, experiments with people standing and walking in front of the camera are complemented. The dataset consists of 14 people, mostly employees, and students from the laboratory.

For the first three datasets, the camera is mounted over a stretcher. The stretcher at the event and the hospital datasets are different. Furthermore, the installation of the sensors did not pay attention to the same height or distance to the stretcher. Therefore, the distance to the stretcher differs between the datasets.

Due to privacy issues, the datasets only contain the depth and the thermal information. The datasets are available via https://osf.io/rhq3m/ [46]. Each frame from the sensors is stored as a point cloud within the common PCD file format, used by the point cloud library [47]. An arbitrary point in the cloud contains the Cartesian coordinates p and three values for color—red, green and blue channel. The data can be enhanced with temperature values *t*.

The name of each frame contains the metadata of each person in front of the camera. The data name is structured as follows GENDER_GROUNDTRUTH_PERSONID_FRAME_ID.pcd. Besides the raw data from the sensors, an already segmented version of each frame exists within the repository. Furthermore, the parameters from intrinsic and extrinsic calibration are available. The authors gratefully acknowledge collaboration and joint work to improve the outcome of body weight estimation based on RGB-D data, especially for the clinical application.

## 5. Experiments and Results

For the upcoming section, the presented algorithm is evaluated for standing and walking people. Experiments for lying people are presented and discussed in the previous work [15,20].

The validate the experiments, different metrics are used for comparison: For each measurement the absolute error *e* can be calculated, having the ground truth value $\hat{x}$ as well as the estimated value $\tilde{x}$ by $e_{i}=\hat{x}-\tilde{x}$. The absolute error would be good to compare a group of people having the same body weight and differ only in their visual appearance. The here presented group of people for testing has a high variety of body weight and visual appearance. Therefore, the absolute error is not sufficient for comparison. Better for comparison of variant datasets is the relative error which is defined for an arbitrary dataset with

(3) $$\epsilon_{=}\frac{{\hat{x}}_{i}-{\tilde{x}}_{i}}{{\hat{x}}_{i}}=\frac{e_{i}}{{\hat{x}}_{i}}$$

Another way to prove and benchmark the body weight estimation approach is the mean absolute error (MAE). The absolute error of each dataset $e_{i}$ is summed up and divided by the total number of datasets for benchmarking. It is defined by

(4) $$e_{mae}=\frac{1}{N}\sum_{i=1}^{N}\left|e_{i}\right|.$$

Further, the mean square error (MSE) can be used for validation. Here the absolute error is squared before summation. It is defined by

(5) $$e_{mse}=\frac{1}{N}\sum_{i=1}^{N}e_{i}^{2}.$$

Compared to the mean absolute error, outliers were weighted stronger.

### 5.1. Standing

In contrast to experiments for people who were lying down, the most correlating feature—the volume—cannot be used because no reference surface for the back of a person exists. Therefore, the body weight estimation has to rely on the remaining features. A previous experiment with the two datasets from a hospital and the event dataset illustrated that the body weight estimation gets worse if the volume is missing. Nevertheless, the decrease in accuracy can be sufficient for other applications.

For the experiment, the dataset W8-300 generated by Nguyen et al. [26] is applied. It contains 299 people standing in front of a Microsoft Kinect camera. The color and the depth frame are saved separately with a resolution for each channel of 8 bit. The segmentation has been done in advance based on ground detection with RANSAC model [40]: The images in the dataset are already segmented, only containing the person’s data as a depth and color image; the background is not visible. The file name of each dataset contains first the gender, second the ground truth body weight, and lastly the surname of the person. The ground truth body weight varies within a range starting from 40 kg up to 104 kg. In the experiments, 202 males and 97 females participated.

**Figure 8:**
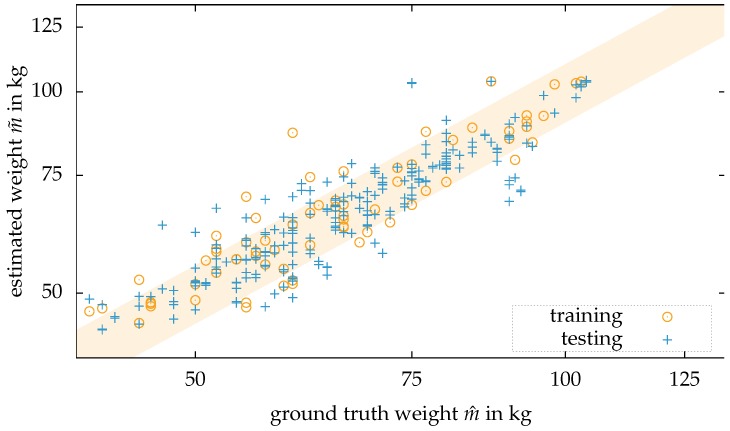
Results of the experiment with people standing in front of the camera based on the here proposed algorithm and the W-300 dataset contributed by Nguyen et al. [26]. The orange area marks the range for the relative error of ±10 percent.

Figure 8 illustrates the result from the dataset: First, the ground truth ordered datasets are shuffled. For training of the ANN, 70 percent of the dataset were used; the other 30 percent were applied for testing.

All people were not told to hold a fixed posture but most of them were standing normally with their arms aside.

Nguyen et al. [26] compared the MAE in their publication: They reached a MAE of 4.62 kg for female and 5.59 kg for male persons. Without the discrimination in gender, the algorithm performs with a MAE of 5.20 kg. This experiment also includes the ground truth of the gender for the applied model. Compared to their results, the here performed experiment reaches an MAE of 4.3 kg. The approach presented by [25] can outperform the here presented results with a MAE of 2.7 kg. However, the sample size in the published article contains only six subjects.

### 5.2. Walking

In addition to the previous experiments, walking people should also be estimated for their body weight. Therefore, a dataset was recorded with students and employees of the Technische Hochschule Nürnberg, Georg Simon Ohm, walking in front of a Microsoft Kinect One. The person is walking towards the sensor, starting at a fixed distance. A marker on the floor shows the limitation of the recorded scene, due to the FOV of the sensor. The sensor is mounted on a tripod in a height of around 1.5 m.

**Figure 9:**
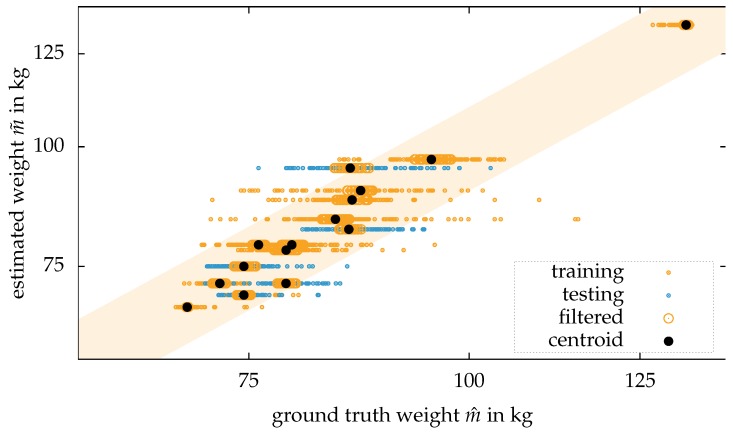
Results of the experiment with people walking towards the camera: The estimations for every frame for an arbitrary person generate a set of estimations, formed as a line together with the ground truth value in the scatter plot. Based on Euclidean clustering, 80 percent of the estimations are removed from the dataset. The final estimation is given based on the centroid of the remaining estimations. The orange area marks the range for the relative error of ±10 percent.

The setting for this experiment is described in detail in the previous section. Figure 9 illustrates the results of this experiment as a scatter plot:

The estimations for an arbitrary person lead in the scatter plot points, aligning on a horizontal line. Often, most of the estimations are outside of the ±10 percent bound. However, some estimations appear to be more dense to other estimations than some outliers. The previously presented approach for clustering now minimizes the set of estimations of an arbitrary person (here marked in bigger points) and calculates the centroid of these sets. For this small sample size of 14 subjects, all of the final estimations were within a range of ±10 percent.

The results provided by Labati et al. [27] outperform the here presented approach when comparing the standard deviation. In contrast to that, the proposed approach outperforms the estimation for walking people presented by Arigbabu et al. [28].

## 6. Discussion

**Figure 10:**
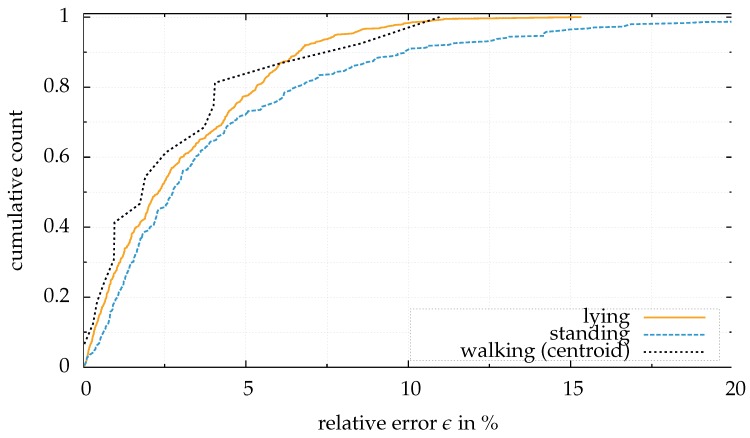
Comparison between the different settings for body weight estimation: Although the results for the estimation with standing people from the W8-300 dataset is the worst in this cumulative plot, the results can be sufficient for a certain applications. Due to the clustering approach, the estimation of walking subjects outperforms the other settings within the range of ±5 percent. The results for the lying patients are taken from Pfitzner et al. [22].

All presented experiments rely on the same set of features. Table 7 compares the result from walking and standing people for body weight estimation: The estimation works best if the subject is lying on a medical stretcher, comparing the results for the relative error and the percentage of in range estimations. This result occurs because in this configuration the volume of the subject can be extracted easily. Further, the variety of posture and position of the subject is low in the overall datasets [22]. The algorithm works with different types of sensors, e.g., a structured light sensor (Kinect) as well as a time of flight sensor (Kinect One). Figure 10 illustrates the relative error in a cumulative plot for lying, standing and walking subjects.

The results for subjects standing in front of a camera are less accurate in nearly every category. However, over 90 percent of the body weight estimation is within a range of ±10 percent. Comparing the here presented approach with the algorithm presented by Nguyen et al. [26], the experiment performs better for the dataset W8-300 with a MAE of 4.31 kg, facing 5.20 kg. In contrast to that, the estimation of subjects walking towards the camera is outstanding. However, the results rely on a small set of subjects. Therefore, the experiment is far from being statistically significant, but it proves the concept.

Although the system with its features is suitable for body weight estimation of lying, standing and walking subjects, there are some limitations. The previously trained ANN can only provide a sufficient result for the body weight estimation when a similar subject has been seen in advance, which is common for machine learning approaches. At a public laboratory event, children were estimated with relative errors in body weight of up to 50 percent—due to not being seen before. The used model was trained with patients from the hospital, where subjects younger than 18 years were excluded in the dataset. While the pose of the subjects lying in the clinical scenario is similar, the pose for walking subjects can vary strongly from frame to frame. For the here presented small experiment, all subjects are facing the camera and walking towards it. In a scenario with the people walking differently, e.g., walking sideways, the algorithms would not provide sufficient estimation results.

## 7. Conclusions and Future Work

This paper presented a novel approach for the estimation of body weight. In contrast to related work, the approach with its feature vector was tested for lying, standing and walking subjects. Experiments proved that the estimation is possible within a given range. The algorithm and the extracted features previously presented in [15] are also able to provide an estimation of standing and walking people. The missing volume—which correlates with the body weight the most [22]—is the reason the estimation for a single frame of a walking subject is worse than for a lying person. However, the estimation on a sequence of frames combined with the presented clustering provides a sufficient body weight estimation. In direct comparison with the approach for body weight estimation approach from Nguyen et al. [26], the approach presented here can outperform the results, while being applied to the same dataset.

For future work, it is the aim of the here presented project to obtain a bigger dataset: The estimation of standing people should be expanded to an approach where people do not need to face in the direction of the sensors. Further, the path for the estimation of walking people should be made more variable so people can move freely in front of the camera. This approach needs a higher demand for varying data. The authors gratefully acknowledge future joint work to improve the outcome of the algorithm and to develop a bigger dataset for experiments and modeling.

## Figures and Tables

**Table 1:** Results for contact-less human body weight estimation from related work in alphabetic order. The results are not directly comparable due to different evaluation metrics.

Method	Sensor	Approach	Constrains	Results
Cook et al. [24]	RGB-D structured light	image processing to reconstruct the volume	sample size 6 subjects	only volume estimation
Pirker et al. [19]	8 stereo cameras	image processing to reconstruct the volume	scene has to be known	only volume estimation
Nguyen et al. [26]	RGB-D structured light	machine learning with l2-regularization and support vector regression		5.2 kg MAE
Velardo and Dugelay [25]	RGB-D structured light	machine learning with multiple regression analysis	sample size 15 subjects	2.7 kg for a single subject
Pfitzner et al. [20]	RGB-D structured light	image processing to reconstruct the volume	person is lying on a flat surface	79.1% within relative error of 10%
Pfitzner et al. [15]	RGB-D structured light	machine learning with ANN	person is lying on a flat surface	89.6% within relative error of 10%
Pfitzner et al. [22]	RGB-D structured light and ToF	machine learning with ANN	person is lying on a flat surface	95.3% within relative error of 10%
Labati et al. [27]	2 RGB cameras	machine learning with ANN	sample size 20 subjects	2.3 kg std error
Arigbabu et al. [28]	RGB cameras	machine learning with ANN		4.66 kg MAE

**Table 2:** Property table of used sensors: The three sensors are selected for the body weight estimation because of their similar FOV, which provides a total view of the patient on the stretcher. For the 3D sensors, the measurement range is sufficient. The frame rate of at least 30 Hz is acceptable, while the thermal camera provides a frame rate of 80 Hz.

Model	Kinect	Kinect One	Optris PI400
Principle	Structured Light	Time-of-Flight	Thermal Camera
Resolution	320×240	512×424	382×288
Field of View	$57^{\circ }\times 43^{\circ }$	$70^{\circ }\times 60^{\circ }$	$62^{\circ }\times 49^{\circ }$
Frame rate	30 fps	30 fps	80 fps
Dimensions	73×283×73 mm${}^{3}$	249×66×67 mm${}^{3}$	46×56×90 mm${}^{3}$
Weight	564 g	1400 g	320 g
Power consumption	12 W	32 W	<2.5 W via USB
Interface	USB 2.0	USB 3.0	USB 2.0
Price	$100	$200	$3500

**Table 3:** Tested hardware including time measurements for the estimation: The biggest part of processing time is used to segment the patient from the environment. In contrast to that, the extraction of the features and the processing via an artificial neural network is small. The total time includes visualization and logging during the processing.

	Desktop Computer	Dell M4800 Mobile Computer	Raspberry PI 3	Asus Tinkerboard
Processor	Intel i7-4820K	Intel i7-4900MQ	ARM Cortex-A53	Rockchip RK3288
Nr. of Threads	8	8	4	4
max. Clock	3.90 GHz	3.80 GHz	1.2 GHz	1.8 GHz
TDP	130 W	47 W	<3.7 W	5 W
Time for Segmentation	239 ms	245 ms	5321 ms	2661 ms
Time for Estimation	22 ms	23 ms	267 ms	212 ms
Total Processing Time	263 ms	270 ms	5604 ms	2885 ms

**Table 4:** List of features for body weight estimation $\forall \;p_{j}\;\in \;P^{P}$. The table further lists the invariance of each feature by scale (s), rotation (r), translation (t), perspective (pe) and posture of the person (po) with + (invariant), 0 (invariant with limitations) and - (not invariant). The equations in the table are taken from the previous work [22].

		Invariance						
	Feature	s	r	t	pe	po	Equation
$f_{1}$	volume	+	+	+	0	-	*v*
$f_{2}$	surface	+	+	+	0	-	*s*
$f_{3}$	number of patient’s points	-	+	+	0	-	$|P^{P}|$
$f_{4}$	density	-	+	+	0	-	$|P^{P}|\;/\;\left|P\right|$
$f_{5}$	1. eigenvalue	+	+	+	0	-	$\lambda_{1}$
$f_{6}$	2. eigenvalue	+	+	+	0	-	$\lambda_{2}$
$f_{7}$	3. eigenvalue	+	+	+	0	-	$\lambda_{3}$
$f_{8}$	sphericity	+	+	+	0	-	λ3∑jλi
$f_{9}$	flatness	+	+	+	0	-	2·(λ2−λ3)∑iλi
$f_{10}$	linearity	+	+	+	0	-	(λ1−λ2)∑iλi
$f_{11}$	compactness	+	+	+	0	-	1n∑i(pj−p¯)2
$f_{12}$	kurtosis	+	+	+	0	-	1n∑j||pj−p¯||
$f_{13}$	alt. compactness	+	+	+	0	-	$\sum_{j}{\left(p_{j}-\bar{p}\right)}^{4}\;/\;f_{9}$
$f_{14}$	distance to person	+	+	+	+	0	*d*
$f_{15}$	contour length	-	+	+	-	-	$l_{c}$
$f_{16}$	contour area	-	+	+	-	-	$a_{c}$
$f_{17}$	convex hull length	-	+	+	-	-	$l_{h}$
$f_{18}$	convex hull area	-	+	+	-	-	$a_{h}$
$f_{19}$	gender	+	+	+	+	+	*g*

**Table 5:** Changes in features with different poses: Five different scenes illustrate the change in feature values depending on the posture.

Features	Scene 1	Scene 2	Scene 3	Scene 4	Scene 5
Surface	9.5 × 10^−1^	9.7 × 10^−1^	9.6 × 10^−1^	8.6 × 10^−1^	9.7 × 10^−1^
Density	1.1 × 10^−1^	1.1 × 10^−1^	1.2 × 10^−1^	1.0 × 10^−1^	1.3 × 10^−1^
1^st^ eigenvalue	4.7 × 10^3^	4.7 × 10^3^	5.1 × 10^3^	4.5 × 10^3^	5.4 × 10^3^
2^nd^ eigenvalue	3.9 × 10^2^	4.6 × 10^2^	6.9 × 10^2^	2.5 × 10^2^	5.2 × 10^2^
3^rd^ eigenvalue	7.1 × 10^1^	3.2 × 10^2^	7.9 × 10^1^	9.4 × 10^1^	7.7 × 10^1^
Sphericity	4.1 × 10^−2^	1.8 × 10^−1^	4.0 × 10^−2^	5.8 × 10^−2^	3.8 × 10^−2^
Flatness	1.2 × 10^−1^	5 × 10^−2^	2.0 × 10^−1^	6 × 10^−2^	1.4 × 10^−1^
Linearity	8.3 × 10^−1^	7.7 × 10^−1^	7.5 × 10^−1^	8.8 × 10^−1^	8.1 × 10^−2^
Compactness	4.6 × 10^−1^	4.6 × 10^−1^	4.6 × 10^−1^	4.7 × 10^−1^	4.5 × 10^−1^
Kurtosis	5.4 × 10^3^	5.5 × 10^3^	6.2 × 10^3^	5.0 × 10^3^	6.0 × 10^3^
AltCompactness	8.6 × 10^−1^	8.7 × 10^−1^	8.6 × 10^−1^	8.6 × 10^−1^	8.7 × 10^−1^
Contour length	1.0 × 10^3^	1.4 × 10^3^	1.4 × 10^3^	1.1 × 10^3^	1.4 × 10^3^
Contour area	2.5 × 10^4^	2.5 × 10^4^	2.8 × 10^4^	2.1 × 10^4^	1.4 × 10^4^
Convex hull length	8.2 × 10^2^	8.3 × 10^2^	9.3 × 10^2^	8.0 × 10^2^	8.8 × 10^2^
Convex hull area	3.0 × 10^4^	3.5 × 10^4^	4.3 × 10^4^	2.6 × 10^4^	3.7 × 10^4^
Distance	1.8	1.8	1.7	1.8	1.6
color	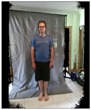	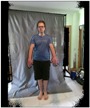	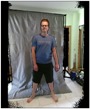	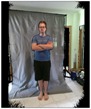	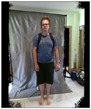
segmented depth	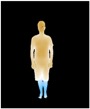	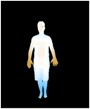	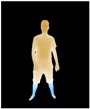	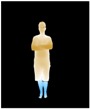	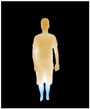

**Table 6:** Datasets applied for this article: The first two datasets are recorded in a trauma room of the University Hospital Erlangen, Germany. The third dataset is based on a public event in a laboratory, containing visitors of this event. The fourth set is recorded with employees and students of the laboratory. For comparison, the average body weight of the German population in 2009 is 73.9 kg [45]. This average value is close to the first three datasets. The last dataset W8-300 is recorded by [26], showing people standing in the front of a Kinect camera.

Dataset	Sensors	Scenario	Real Weight in kg				Gender		Total
			min	max	Mean	σ	Female	Male	
HospitalNoThermo	K	lying	48.8	165	78.3	17.3	93	99	192
Hospital	K, T	lying	48.6	129	77.8	17.1	72	55	127
Event	K, K2,T	lying	48.8	114	78.6	12.0	24	82	106
Walking	K2	walking	68	134	84.2	16.4	0	14	14
W8-300 [26]	K1	standing	40	104	67.2	14.7	97	207	299

**Table 7:** Results from experiments for standing and walking people. Additionally, the results from Pfitzner et al. [22] are added for comparison. The lower part of the table illustrates the results from related work, when available in detail. The best result is marked in bold for each category.

	Dataset	Size	Relative Error in %				In Range in %			Error in kg/${\mathrm{kg}}^{2}$	
			min	max	Mean	σ	5	10	20	MAE	MSE
Lying [22]	Event	106	−8.7	14.3	0.90	4.80	75.6	95.3	**100**	2.86	**13.8**
Standing	W8-300	299	−28.8	16.76	**−0.1**	5.80	70.5	91.3	99.3	4.31	33.5
Walking	Walking	14	**−6.7**	**9.38**	0.32	3.88	**78.5**	**100**	**100**	3.30	20.5
Nguyen et al. [26]	W8-300	299								5.2	
Velardo and Dugelay [25]		6			3.6					**2.7**	
Labati et al. [27]		20				**2.3**					
Arigbabu et al. [28]		13								4.66	

## Abbreviations

The following abbreviations are used in this manuscript:

ANN	artificial neural network
FPV	field of view
IR	infrared
MAE	mean average error
MSE	mean square error
RANSAC	random sample consensus
RGB	red green blue
RGB-D	red green blue depth
RGB-D-T	red green blue depth thermal
PCD	point cloud data
rtPA	recombinant tissue plasminogen activator
TDP	thermal design power
ToF	time of flight

References
